# Moral Challenges of Nurses and Volunteers in Dutch Palliative Care. A Qualitative Study

**DOI:** 10.1177/08258597221098129

**Published:** 2022-05-25

**Authors:** Geerke van den Bosch, Malene van Schaik, H. Roeline Pasman, Rien Janssens, Guy Widdershoven, Suzanne Metselaar

**Affiliations:** Ethics, Law and Humanities, Amsterdam, the Netherlands

**Keywords:** palliative care, ethics, ethics support, moral challenges, nurses, volunteers

## Abstract

**Objective:** To identify moral challenges experienced by nurses and volunteers in palliative care. **Methods:** A qualitative hermeneutic research design was used. Interviews with nurses (*N* = 10) and volunteers (*N* = 4) working in palliative care, in-home care, and hospice setting. Participants were recruited through maximum variation, a purposive sampling technique. Transcriptions were analyzed using qualitative thematic content analysis and open coding. **Results:** Two themes were identified, each with three subthemes: theme (A) Moral challenges regarding organizational and professional aspects contained the subthemes (1) dealing with protocols and regulations, (2) different professional perspectives on good care, and (3) limits of professionalism. Theme (B) Moral challenges regarding the patient and their family members contained the subthemes (1) dealing with the patient's wishes, (2) the patient's wish to die, and (3) dealing with family members. **Conclusion:** Nurses and volunteers working in palliative care are confronted with a wide range of moral challenges. Insight into ‘real-world ethical challenges’ of healthcare providers is important to provide adequate support to nurses and volunteers working in palliative care.

## Introduction

Providing palliative care has been described as fulfilling, both for nurses and volunteers. Nonetheless, it is also experienced as emotionally taxing.^1,2^ Palliative care involves intense human interactions in a period where patients and those close to them must face the end of life. Palliative care in the Netherlands involves nurses and volunteers working side by side in hospices, care homes, and home care settings. Whereas professional caregivers focus on the medical and technical aspects of care, volunteers provide emotional and existential support to patients and family members.^3^ Both volunteers and health care professionals are regularly confronted with similar situations they experience as (morally) troublesome or challenging.^4–7^

Moral challenges (dilemmas, questions, doubts) arise from situations in which people experience an internal conflict between the norms and values that (implicitly) guide them in their work.^8^ Furthermore, moral challenges are experienced when healthcare professionals are involved in situations where there is a disagreement on what is ‘good care’, or what healthcare professionals see as ‘good care’ is not in line with institutional practice, available resources, laws, or guidelines. In palliative care specifically, moral challenges arise from situations with differing perspectives on the continuation of life-prolonging interventions. This goes from having to choose between adhering to protocols or diverging from them to meet the wishes of the patient or their family members near the end of life, to issues around euthanasia and palliative sedation.^4,9^

Knowledge of the specific moral challenges caregivers in palliative care struggle with – in this case, nurses and volunteers – is important to provide them with adequate support in dealing with these moral challenges.^9^ Therefore, the purpose of this study was to gain more insight into the moral challenges that nurses and volunteers face in current palliative care practice in the Netherlands. Correspondingly, our main research question was: ‘Which types of moral challenges are experienced by nurses and volunteers in palliative care practice in the Netherlands?’.

## Methods

### Study Design

A qualitative hermeneutic design^10,11^ was used to obtain an in-depth understanding of the moral challenges experienced by participants and their interpretation of these challenges. Semi-structured interviews with nurses and volunteers working in palliative care took place.

### Setting and Participants

The study was carried out in Amsterdam, the Netherlands. Participants were working in a home care setting (n = 7 nurses), hospice setting (n = 3 nurses, n = 1 volunteer), or an organization that matches volunteers with terminally ill patients to provide (psycho)social support (n = 3 volunteers). Maximum variation sampling, a purposive sampling technique,^11^ was used to include participants that could provide us with diverse examples of moral challenges in palliative care. It generated a variety of volunteers and professionals working in either an extramural setting (home care) or an intramural setting (hospice or palliative unit of a care home). Among the participants were both nurses with and without specialized training in palliative care. In line with a qualitative research design, we aimed to capture and describe central themes, rather than reach generalizability.^10^ The participants were recruited in several organizations by contracting executives. They were approached by email or phone. All participants met the following criteria: (1) nurses /volunteers working in palliative care; (2) willing to participate in the study; (3) volunteers trained to work in palliative care; (4) working experience of a year or longer in palliative care. All participants that were approached were willing to participate. Volunteers play a vital role in front-line palliative care provision and work closely together with nurses, as both deliver bedside care.^3,12^ They take up a specific role in relation to the patient and family members, through their caring activities focused on supportive, psychosocial, and existential care. By including both perspectives, we expected to gain a broad and detailed insight into the moral challenges that may be experienced in palliative care.

### Data Collection

One-on-one semi-structured interviews were carried out using an interview guide (Table 1). Participants were encouraged to reflect on their values, speak at full length and in full detail.^13^ After the first three interviews, the researchers concluded that there was no need to revise the topic list. Interviews were tape-recorded and transcribed verbatim. We concluded that data saturation was reached after no new themes emerged during analysis of the last couple of interviews.

**Table 1.** Topic List Semi-Structured Interviews.

Start interview: – Explanation about the aim of the study – Practical aspects: Guarantee confidentiality Member check Time available Signing informed consent paper – Explanation about the characteristics of moral challenges and the differences between values and norms
General information: – Biographical background: Education Career history Current job in palliative care: since when? Work experience?
Open-ended questions: – Which types of moral challenges do you experience in your work? – Can you give some specific cases? – What makes this moral challenging for you? – Which values are at stake in this moral challenge and why?

### Data Analysis

We used thematic analysis and open coding as an inductive approach.^11^ The method was as follows: (1) transcription of the interviews verbatim; (2) familiarizing with the data; (3) identifying codes and themes; (4) coding the data; (5) organizing codes and themes. The first four interviews were analyzed by three researchers independently after which the analysis was discussed in the broader project group. Differences in codes were discussed until consensus was reached. No subgroup analysis was conducted because of the small sample size.

### Qualitative Measures

All 14 participants were invited to respond to the written member check report which increases this study’s credibility and trustworthiness.^11^ Four participants gave clarifications and three participants recalled additional experienced moral challenges after the interview. All clarifications and additions were added to the data and included for analysis of this study. Furthermore, theoretical triangulation was obtained through relevant literature which increases the credibility of this study.^14^ As discussed before, researcher triangulation was obtained through individual analyses of the first four interviews after which the analysis was discussed in the broader group until consensus was reached on the codes. Research triangulation during the analysis phase increases the validity and reliability of this study.^15^

### Ethical Considerations

Prior to inception of the study the protocol was submitted to the Medical Ethics Review Committee of VU University Medical Center, IRB00002991, which declared that according to Dutch law ethical review was not needed.

If permission was obtained for the interviews, the study was explained in depth, and written informed consent was received.

## Results

Participant characteristics are shown in Table 2. The interviews varied in length from 36 to 76 minutes. Two main themes were identified and within each theme, six sub-themes were identified as presented in Table 3.

**Table 2.** Participant Characteristics.

Function	Specialization	Setting	Sex	Years of work experience
Nurse 1		Palliative team in home care	Female	25-30 years
Nurse 2		Home care	Female	45-50 years
Nurse 3		Home care	Male	5-10 years
Nurse 4		Home care	Male	20-25 years
Nurse 5	Case manager dementia	Home care	Female	25-30 years
Nurse 6		Hospice	Female	20-25 years
Nurse 7	Nurse specialist in palliative care	Hospice	Female	30-35 years
Nurse 8		Palliative team in home care	Female	15-20 years
Nurse 9		Palliative care unit in a nursing home	Female	15-20 years
Nurse 10	Nurse specialist in palliative care	Hospice	Female	20-25 years
Volunteer 1		Volunteering at people's homes through a volunteer's organization	Female	1-5 year
Volunteer 2		Volunteering at people's homes through a volunteer's organization	Female	5-10 years
Volunteer 3		Hospice	Female	1-5 years
Volunteer 4		Volunteering at people's homes through a volunteer's organization	Female	1-5 years

**Table 3.** Themes and sub-Themes.

Theme	Sub-theme
Moral challenges regarding organizational and professional aspects Moral challenges regarding the patient and their family members	Dealing with protocols and regulations Different professional perspectives on good care Limits of professionalism Dealing with the patient's wishes The patient's wish to die Dealing with family members

## Moral Challenges Regarding Organizational and Professional Aspects

### Dealing with Protocols and Regulations

Both nurses and volunteers indicated that, in certain situations, protocols and regulations presented them with moral challenges, as they felt they were obstructing good care in some way. For instance, a participant described how they wanted to put up the bedrails to prevent a patient from falling out of bed while the patient was unable to give permission. However, the protocol states that the bedrails are only to be put up if the patient gives permission, even though there was a realistic risk of the patient falling out of bed. The participant felt a tension between adhering to the protocol and respecting their patient's autonomy and freedom (ie, not being restrained), versus the wish to keep their patient safe and prevent him from harm:

“We are not allowed to put the bedrails up without his permission, but we put them up for his safety. But by putting them up, we restrict him in his freedom.” [Nurse #8]

Volunteers reported similar concerns. Challenges are especially encountered in situations where a patient requests help, but volunteers lack the qualification. The quote below demonstrates how a volunteer felt tension between following the guidelines versus adhering to the patient's request and chose the latter:

“What do you do when someone asks you for help? You are not qualified; hence, you are not allowed to help. [The patient] asked me if I would help him to use the toilet. I appreciated that he trusted me, so I helped him.” [Volunteer #4]

Both nurses and volunteers may experience challenges regarding protocols and regulations if they feel they are being limited in delivering good care to patients and tend to give priority to values such as attentiveness over (strictly) following protocols or guidelines.

### Different Professional Perspectives on Good Care

Moral challenges arising from different perspectives on good palliative care often revolved around decisions concerning either continuing or withdrawing curative treatment. These different perspectives were specifically mentioned by nurses in the context of their collaboration with physicians. Nurses sometimes felt the physician did not see the ‘bigger picture’, ie, the practical consequences of medical decisions for the patient's (quality of) life at its final stage.

“We went to the dermatologist and the advice was to operate [the skin cancer] (…) The patient is diagnosed with severe dementia. Operating will (…) lead to hospitalization and lesions. Is that good care for this patient?” [Nurse #5]

Nurses stressed the importance of comfort in the last stage of life, whereas they feel physicians are too focused on curative treatment to extend life:

“They are not so much aimed at comfort. GPs are very much aimed at curing.” [Nurse #3]

According to the nurses in this study, different professional perspectives on good care can lead to suboptimal care for their patients and cause feelings of stress among participants.

### Limits of Professionalism

Value conflicts may arise between acting professionally and keeping professional distance versus proximity and compassion. In general, participants mentioned that the palliative stage of life can be very emotional for patients and family members, but also for caregivers themselves. Participants expressed doubts in this regard:

“I sometimes cry [in the presence of the patient]. Is that a bad thing?” [Nurse #1]

Struggles with finding the balance between compassionate care and not burdening the patient with one's emotions are mentioned:

“A colleague often says: ‘It's a patient, not my partner or family’. I realize that; I do not have the idea that [patients] are my friends, but I do feel involved. However, I do not show it to the patient. I think professional behavior is very important. But it does upset me.” [Nurse #2]

Volunteers also expressed doubts regarding finding a balance between ‘proximity’ and ‘professional distance’. For instance, they were in doubt about whether to go to a patient's funeral and how to manage expectations about their involvement.

“In the last days of his life, [the patient] called me a good friend. I really liked him, but a good friend means something else to me. […] Did I deceive this man? Did I give false or wrong ideas about myself and my involvement?” [Volunteer #4]

The fact that their role is less well-defined and therefore more ambiguous than the role of nurses, might play a part in these moral doubts of volunteers.

## Moral Challenges Regarding the Patient and Their Family Members

### Dealing with the Patient's Wishes

The first sub-theme of the second theme concerns dealing with the patient's wishes. Especially if these wishes go against what the caregiver considers good care, this can lead to moral challenges between respecting the patient's autonomy and promoting what is seen as best for the patient. For instance, if a patient refuses morphine for pain relief, based on assumptions about side-effects:

“Wishes of patients, how do you honor them? Sometimes people suffer a lot (…) but they fear the use of morphine. You can give them information or ask them to try it. But if people say, ‘I do not want it; I am afraid of it’, then you feel powerless as a nurse because you see people suffer while you know there's another way. But that has to do with autonomy.” [Nurse #10]

Acceptance of death is seen as important for the patient's and family's wellbeing by both nurses and volunteers. However, sometimes patients held off conversations on the topic, which made caregivers unable to act in accordance with their own values, ie having conversations about the patient's impending death, considering the importance of acceptance.

“There are situations in which patients cannot accept that they are dying. (…) It creates a conflict within me if I see that people struggle with letting go and accepting that life is finite.” [Nurse #10]

Finally, nurses experienced a lack of resources making it impossible to fulfill the patient's wishes. Feelings of responsibility towards the patient may conflict with responsibility for (not overburdening) the staff:

“The transfer nurse of the hospital called: a patient wanted to die at home. Although you’d wish for everyone to die at home, you think: ‘How many nurses do we have right now?’. (…) Then you decide that it's not possible because you do not want to overburden your team. It was just before the weekend, so you prevented a lot of misery for the team. Nonetheless, it gnaws at you because you did not honor someone's wish to die at home.” [Nurse #4]

Overall, participants felt the importance of meeting the patient's wishes as a key element of good palliative care, especially in the dying phase. Granting a patient's last wish is considered a particularly heavy responsibility:

“In end-of-life-care, there is just one chance to do it right.” (Nurse #2)

### The Patient's Wish to Die

Some patients have a strong wish to die and request physician-assisted dying or euthanasia (which, if strict conditions are met, is legal in the Netherlands). Challenges related to euthanasia, at least in specific situations, were frequently expressed by nurses, although it is not their task to decide on granting a euthanasia request - as this is the responsibility of physicians in the Netherlands. Some participants were hesitant about euthanasia but respected the patient's wish.

“I am against euthanasia, but it is not my choice, so I try to separate it [own values and norms from the patients’ choice]. I am present at the moment it happens, to support the patient.’ [Nurse# 9]

However, this nurse, as well as other participants, mentioned that sometimes, they had serious difficulties with the choice for euthanasia:

“The last patient who requested euthanasia had been healthy his whole life, but now he had difficulties with accepting that he was ill. I thought [the euthanasia] was too soon. I did not attend, emotionally I couldn't.” [Nurse #9]

Volunteers experienced similar moral challenges. One volunteer was against euthanasia because of religious beliefs. She experienced a value conflict between compassion for the patient versus acting in line with her own values:

“Does my opinion count? Out of compassion I could be present and support the patient. But maybe that goes too far for me. No, I could not be a witness of it [the euthanasia].” [Volunteer #3]

## Dealing with Family Members

Participants mentioned that attuning to the needs of family members is an important aspect of good palliative care:

‘The patient will die, but the family lives on. So, I want it to go as well as possible for them’. [Nurse #9]

The process of letting a beloved family member go is hard. This may lead to difficult situations for caregivers: to what extent are they responsible for the needs of family members when this compromises good care for the patient? A volunteer gives an example of a son who could not (yet) accept the palliative state his mother was in:

“The son is very fond of his mom; he does not want to miss her yet. (…) She is 94 and has fallen out of bed, dragged to the hospital to see if anything was broken. [He] says to me: ‘If anything would have been broken, she probably should’ve been operated.’ And I think: ‘operating…?’’ (…) I know that it's not my responsibility, but I do have an opinion about the way in which he's trying to keep her alive. I once tried to discuss this, but I noticed I have to be careful.” [Volunteer #1]

Communication and good relationships between loved ones are highly valued. Therefore, nurses and volunteers are actively seeking ways to improve disrupted family relationships:

“The patient was one piece of anger, frustration, and distrust towards his family, and us. We decided that he needed medication to calm down (…). Hopefully, it would bring back trust to improve the relationship with his family. (…) [Eventually] I got him to accept medication and he became calm. (…). Connecting with people you love, is very important to me in the final stage.” [Nurse #10]

This nurse stressed the importance of good relationships in the last phase of life and, consequently, tried to improve family relations.

## Discussion

This study provides an overview of moral challenges among nurses and volunteers in palliative care. It presents some major moral issues which further corroborate findings from the literature.

The first theme, moral challenges related to a professional role, refers to the moral challenges participants experienced regarding dealing with protocols and guidelines, with different professional perspectives on good care and professional behavior. Participants mentioned that delivering good palliative care entails more than merely following protocols. Protocols and guidelines can cause moral doubts when it prevents participants from acting in accordance with their own values.^16^ According to nurses, physicians were often more inclined towards (continuing) curative treatment, whereas nurses were more inclined towards focusing on comfort and quality of life, which is in line with previous studies.^17^ Divergences in the perspectives of physicians and nurses have been described as a source for moral challenges and distress.^18–21^ Finding a balance between proximity and (professional) distance was important for many participants, but specifically for volunteers. This can be explained by the not as well-defined boundaries within their role.^22^

The second theme, moral challenges regarding the patient and family members, demonstrated challenges regarding dealing with the patient's wishes, such as refusal of pain treatment. Professionals experience a lack of confidence in communication skills in this regard,^6^ as it can be difficult to talk with patients about existential needs and dying.^23,24^ Our results emphasize that nurses and volunteers express the need to talk about existential needs and dying, but experience barriers trying to do so. For instance, when a patient cannot accept their impending death. Finally, caregivers experienced a lack of resources, which made it impossible to fulfill the patient's wishes. This is in line with other studies which show that time pressure and personnel shortages may lead to morally troublesome situations and hinder nurses in delivering high-quality palliative care.^21,25,26^ Euthanasia is mentioned regularly by participants as a cause for moral challenges and distress. This is consistent with existing literature.^27^ Finally, balancing between the competing needs of family members and a patient's needs involves challenges for both nurses and volunteers.^28,29^

## Clinical Implications

Insight into the moral challenges of healthcare providers is important for providing adequate support.^9^ The Dutch Quality Framework on Palliative Care states that “healthcare providers and volunteers [are expected to be able to] recognize moral challenges. (…) [And] be sensitive to context and relationships in which moral questions arise and are capable to reflect on these questions with others and from various perspectives”.^30^ Our study shows that our participants were proficient in identifying moral challenges in practice. For dealing with moral challenges adequately, moral competences are needed; not only recognizing moral challenges but also reflecting on these challenges from different perspectives and finding a well-considered way to deal with these situations.^31–34^ Ethics support, such as moral case deliberation, has proven to be supportive to healthcare providers in finding ways to deal with tensions and challenges, by reflecting on perspectives of persons involved and sharing experiences and views.^35^ As a recent study shows, nurses in palliative care often encounter a broad range of moral challenges, while many of these are not represented in ethics training courses.^9^ Furthermore, our findings indicate that volunteers are confronted with moral challenges as well, nonetheless, they receive even less or no training in ethics, nor dealing with moral challenges.^3^ Therefore, future research should focus on how moral competences of nurses and volunteers working in palliative care may be strengthened to deal with these challenges adequately, and how ethics support can reinforce the moral competences of nurses and volunteers.^36^

## Limitations

The sample of our study is varied due to maximum variation sampling, delivering rich data with a broad scope of overarching themes. However, we cannot be certain data saturation was reached, as we only interviewed one nurse from a nursing home and our sample does not include novice nurses. Novice nurses may have provided additional data as it is known that they experience high levels of moral distress.^37,38^ Furthermore, we only included four volunteers. Therefore, we cannot make strong statements on differences between nurses and volunteers. While nurses and volunteers are confronted with similar challenges as they both deliver essential bedside care,^3,12^ volunteers likely experience some (sub)themes of moral challenges differently. For instance, the subtheme ‘communication with physicians’ was expressed less by volunteers than by nurses, because volunteers are not involved in the medical decision-making process. A follow-up study with a bigger sample size would be useful to conduct a more detailed analysis of the differences between these two perspectives. This could result in different needs regarding ethics support.

## Conclusion

Nurses and volunteers working in palliative care are confronted with various moral challenges. We identified two main themes with three sub-themes each. The first theme, moral challenges regarding organizational and professional aspects, includes ‘Dealing with protocols and regulations’, ‘Different professional perspectives on good care’ and ‘Limits of professionalism’. The second theme, moral challenges regarding the patient and their family members, includes ‘Dealing with the patient's wishes’, ‘The patient's wish to die’, and ‘Dealing with family members’. Insight into the experience of healthcare providers is important for providing adequate support to nurses and volunteers working in palliative care and to strengthen their moral competences.
